# Acylated Anthocyanins From Black Carrots and Their Related Phenolic Acids Diminish Priming and Activation of the NLRP3 Inflammasome in THP‐1 Monocytes

**DOI:** 10.1002/mnfr.202400356

**Published:** 2024-10-19

**Authors:** Inken Behrendt, Katharina Becker, Christof Björn Steingass, Ralf Schweiggert, Gabriela Michel, Elvira Friedrich, Daniela Grote, Zoe Martin, Hanna Pauline Dötzer, Mathias Fasshauer, Martin Speckmann, Sabine Kuntz

**Affiliations:** ^1^ Institute of Nutritional Science, Department of Nutritional Science Justus‐Liebig‐University Giessen Giessen Germany; ^2^ Chair of Analysis and Technology of Plant‐based Foods – Focus on Beverages Department of Beverage Research Geisenheim University Geisenheim Germany; ^3^ Institute for Clinical Immunology, Transfusion Medicine and Hemostaseology, Department of Medicine Justus‐Liebig‐University Giessen Giessen Germany; ^4^ Flow Cytometry Core Facility Department of Medicine Justus‐Liebig‐University Giessen Giessen Germany

**Keywords:** anthocyanin‐rich black carrot extract, ASC specks, NLRP3 inflammasome, phenolic acids

## Abstract

**Scope:**

Excessive activation of the nucleotide‐binding oligomerization domain‐like receptor pyrin domain‐containing protein 3 (NLRP3) inflammasome contributes to chronic inflammation. Thus, targeting NLRP3 inflammasome activation by anthocyanins may prevent inflammatory diseases. Therefore, the present study determines the influence of a black carrot extract (BCE) with high amounts of acylated anthocyanins and their related phenolic acids on the NLRP3 inflammasome.

**Methods and results:**

THP‐1 monocytes are pretreated with a BCE, cyanidin‐3‐glucoside (C3G), or hydroxycinnamic acids. NLRP3 inflammasome assembly is initiated by priming THP‐1 monocytes with lipopolysaccharide and/or activating the NLRP3 inflammasome with nigericin. Flow cytometry is used to assess apoptosis‐associated speck‐like protein containing a caspase recruitment domain (ASC) speck formation, as well as ASC and NLRP3 protein expression. Caspase‐1 activity is measured using a bioluminescent assay, and cytokine concentrations are determined by enzyme‐linked immunosorbent assays (ELISA). C3G and phenolic acids diminish ASC and NLRP3 protein expression. In addition, C3G and phenolic acids attenuate ASC speck formation. Furthermore, the BCE and C3G decline caspase‐1 activity. Consistently, IL‐1β and IL‐18 secretion are reduced upon NLRP3 inflammasome activation.

**Conclusion:**

The present study shows that a BCE with high amounts of acylated anthocyanins and their related phenolic acids diminish priming and activation of the NLRP3 inflammasome in THP‐1 monocytes.

## Graphical abstract

Assembly of the NLRP3 inflammasome results in the formation of a supramolecular ASC speck and autoproteolytic activation of caspase‐1, which in turn catalyzes the maturation and release of proinflammatory cytokines. Excessive NLRP3 inflammasome activation contributes to obesity‐induced chronic low‐grade inflammation. The present study shows that acylated anthocyanins from black carrots and their related phenolic acids diminish the priming and activation of the NLRP3 inflammasome in THP‐1 monocytes.

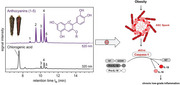

## 1 Introduction

Inflammasomes are part of the innate immune system and sense endogenous and exogenous stress signals which lead to a fast inflammatory response.^[^ ^1^, ^2^ ^]^ The nucleotide‐binding oligomerization domain‐like receptor pyrin domain‐containing protein 3 (NLRP3) inflammasome is the most studied and therefore the best‐characterized inflammasome.^[^ ^3^ ^]^ This multimeric protein complex is expressed in myeloid lineage cells and consists of the sensor protein NLRP3, the adaptor protein apoptosis‐associated speck‐like protein containing a caspase recruitment domain (ASC), and the effector protein caspase‐1.^[^ ^4^ ^]^ Assembly of the NLRP3 inflammasome results in the autoproteolytic activation of caspase‐1, which in turn catalyzes the maturation and release of proinflammatory cytokines.^[^ ^5^ ^]^ Emerging evidence indicates that excessive activation of the NLRP3 inflammasome contributes to obesity‐induced chronic low‐grade inflammation.^[^ ^6^, ^7^ ^]^ Furthermore, inhibition of the NLRP3 inflammasome exerts beneficial health effects in several inflammatory disease models while the host immune response is hardly affected.^[^ ^5^ ^]^ Therefore, many studies have investigated the potential of pharmacological NLRP3 inhibitors for their use as antiinflammatory agents. However, none of these newly identified NLRP3 inflammasome inhibitors has been approved by the food and drug administration so far.^[^ ^3^ ^]^ Consequently, identifying food components targeting NLRP3 inflammasome activation may be a safe and effective strategy to prevent the initiation and progression of inflammation‐related diseases.

Anthocyanins are glycosylated phenolic plant metabolites imparting red, purple, and blue colors to many fruits and vegetables. Their aglycons consist of two phenolic rings (A‐ and B‐ring) which are linked by an O‐heterocyclic C‐ring. Their hydroxyl groups can be substituted with one or several sugars or methyl residues (i.e., forming methoxy groups). The glycosyl moieties in turn may be acylated with aliphatic (e.g., malonic, acetic, and malic acid) or phenolic acids (e.g., ferulic, *p*‐coumaric, sinapinic, and gallic acid).^[^ ^8^ ^]^ So far more than 700 naturally occurring anthocyanins have been identified, with cyanidin derivatives being the most abundant anthocyanins.^[^ ^8^, ^9^ ^]^ Berries such as blueberries and blackberries, as well as pigmented vegetables and tubers such as black carrots, are anthocyanin‐rich sources.^[^ ^10^, ^11^, ^12^ ^]^ While blueberries contain a broad spectrum of nonacylated anthocyanins based on various aglycons,^[^ ^13^, ^14^ ^]^ carrot anthocyanins mainly comprise cyanidin derivatives.^[^ ^15^, ^16^ ^]^ In addition, in black carrots more than 60% of the total anthocyanin levels are represented by derivatives acylated with hydroxycinnamic acids such as ferulic acid (FA), *p*‐coumaric acid (CA), and sinapinic acid (SA).^[^ ^15^, ^16^ ^]^ Recent studies suggest that due to the complex structural properties of acylated anthocyanins, the biological effects may differ from those of their nonacylated counterparts.^[^ ^17^ ^]^ Nevertheless, the extent to which these different effects are associated with a health‐promoting status remains to be investigated.^[^ ^17^ ^]^ Although acylated anthocyanins could be absorbed intact in the gastrointestinal tract, their bioavailability has been described to be poor.^[^ ^14^, ^15^, ^18^ ^]^ However, acylated anthocyanins, which are not absorbed in the small intestine, could reach the colon, where they are broken down by the human gut microbiota into small molecular phenolic acids, both resulting from the cleavage of the acyl groups and the breakdown of the anthocyanidins. In this context, it has already been shown in vitro that pelargonidin sophorosides, which were acylated with hydroxycinnamic and/or malonic acid, can be degraded to 4‐hydroxybenzoic acid and hydroxycinnamic acids.^[^ ^19^ ^]^ Phenolic acids, which are released from acylated anthocyanins, such as caffeic, ferulic, *p*‐coumaric, and vanillic acid as well as phenolic acids, which are degradation products of aglycons such as protocatechuic acid, may be absorbed in the lower gastrointestinal tract and were further metabolized to glucuronidated or sulfated conjugates.^[^ ^11^, ^20^, ^21^ ^]^ In this context, a human placebo‐controlled, cross‐over study showed that urinary excretion of hydroxycinnamic acids increased after consumption of a study meal supplemented with a purple potato extract, which was rich in monoacylated anthocyanins.^[^ ^20^ ^]^ Therefore, phenolic acids may at least partly contribute to the observed antiinflammatory effects of acylated anthocyanins. However, the protective role of acylated anthocyanins from black carrots and their related phenolic acids to prevent chronic low‐grade inflammation by attenuating NLRP3 inflammasome activation in human monocytes has not been explored so far. Therefore, the primary goal of the present study was to determine whether a black carrot extract (BCE) being rich in acylated anthocyanins can influence the activation of the NLRP3 inflammasome in THP‐1 monocytes. In addition, we also aimed to determine the influence of cyanidin‐3‐glucoside (C3G) and phenolic acids, which are components of the acylated anthocyanins in the BCE, on NLRP3 inflammasome activation.

## 2 Experimental Section

### 2.1 HPLC‐DAD and HPLC‐DAD‐ESI‐QTOF‐HR‐MS/MS Analyses of the Anthocyanin‐Rich Black Carrot Extract

A commercially available, anthocyanin‐rich BCE powder was purchased from Döhler (Darmstadt, Germany). According to the manufacturer, anthocyanins were isolated from black carrot juice using an adsorber resin following spray‐drying. An aliquot of 10 mg of the extract was dissolved in 100 mL eluents A and B (1:1 and v/v), filtered through a 0.2 µm syringe filter (PTFE, Macherey‐Nagel, Düren, Deutschland) into glass vials prior to HPLC analyses.

Anthocyanins and further phenolic compounds were analyzed with an Ultimate 3000 UHPLC (Thermo Scientific, Waltham, MA, USA) equipped with a diode array detector (DAD) and a C18 reversed‐phase column (Luna C18 Phenomenex, 150 × 2 mm i.d., particle size 3 µm) operated at an oven temperature of 40 °C. Eluent A was composed of a mixture of H_2_O and formic acid (95:5, v/v), eluent B was pure methanol. The eluent gradient was as follows: 90% A isocratic (1 min), 90%–50% A (12 min), 50%–0% A (12.5 min), 0% A isocratic (16.5 min), 0%–90% A (17 min), and 90% A isocratic (21 min). Total run time was 21 min at a flow rate of 0.25 mL min^−1^. The injection volume was 4 µL. Identification was performed by an Elute UHPLC system coupled to a *tims*TOF Pro 2 quadrupole time‐of‐flight high‐resolution tandem mass spectrometer (Bruker Daltonik, Bremen, Germany) equipped with an electrospray ionization source, utilizing the aforementioned elution program. The obtained UV/vis and mass spectral data were compared to those described previously in the literature.^[^ ^22^ ^]^ Chlorogenic and caffeic acid were identified by comparing retention times as well as UV and mass spectral data to those of authentic reference standards (Carl Roth GmbH + Co. KG, Karlsruhe, Germany), which were also used for quantification in analytical duplicates by HPLC‐DAD applying linear external calibration curves. Chlorogenic acid (calibration range 0.01–100 mg L^−1^) and caffeic acid (0.1–100 mg L^−1^) were detected at 320 nm. An authentic standard of C3G (Carl Roth GmbH + Co. KG) was used for the quantification of anthocyanins (0.25–100 mg L^−1^) at 520 nm. The mass concentration in mg anthocyanin per g of extract was calculated from the C3G equivalents obtained using molecular weight correction factors by analogy to Gras et al. (2015).^[^ ^22^ ^]^ The limit of detection (LOD: 3.3 *σ*/*S*) and quantification (LOQ: 10 *σ*/*S*) expressed in nanogram on the column were deduced from the standard deviation (*σ*) and the slope (*S*) of the calibration curves. The linearity of the calibration curves was assessed using Mandel's test (LOD/LOQ ICH Guideline (www.fda.gov/regulatory‐information/search‐fda‐guidance‐documents/q2r1‐validation‐analytical‐procedures‐text‐and‐methodology‐guidance‐industry).

### 2.2 Preparation of the Treatment Solutions

The treatment solution comprising the powdered BCE was freshly prepared each time by dissolving in cell culture media at 1 mg mL^−1^ followed by sterile filtration. Stock solutions of C3G (purity ≥ 97%; Carl Roth GmbH + Co. KG), FA (4‐hydroxy‐3‐methoxycinnamic acid; purity ≥ 98%; Th. Geyer GmbH & Co. KG, Renningen, Germany), CA (4‐hydroxycinnamic acid; purity ≥ 98%; Carl Roth GmbH + Co. KG), and SA (3,5‐dimethoxy‐4‐hydroxycinnamic acid; purity ≥ 98%, Carl Roth GmbH + Co. KG) were prepared in dimethyl sulfoxide (DMSO; Merck KGaA, Darmstadt, Germany) at 10 mm and stored at −20 °C. Working solutions of all treatments were made in cell culture media.

### 2.3 Determination of Cell Viability and Cytotoxicity by Flow Cytometry

Cell viability and cytotoxicity were assessed by flow cytometry. Therefore, THP‐1 monocytes (2  ×  10^5^ cells well^−1^) were seeded in 48‐well plates and incubated with different concentrations of the BCE (from 15 to 500 µg mL^−1^), C3G (from 3.9 to 125 µm), FA (from 3.9 to 125 µm), CA (from 3.9 to 125 µm), or SA (from 3.9 to 125 µm) at 37 °C and 5% CO_2_ for 24 h. Then, cells were incubated with Guava ViaCount Reagent (Merck KGaA). Cell viability was assessed by flow cytometry on a Guava Muse Cell Analyzer (Merck KGaA) and the percentage of viable cells was quantified as recently published.^[^ ^23^ ^]^

### 2.4 Cell Culture and NLRP3 Inflammasome Activation

The human monocytic cells line THP‐1 (ACC16), which was originally derived from a 1‐year‐old patient with acute monocytic leukemia,^[^ ^5^, ^24^ ^]^ was purchased from Leibniz Institute DSMZ‐German Collection of Microorganisms and Cell Cultures (Braunschweig, Germany). THP‐1 monocytes were maintained in Roswell Park Memorial Institute (RPMI) 1640 GlutaMAX™ medium (Invitrogen GmbH, Darmstadt, Germany) supplemented with 7.5% v/v heat‐inactivated fetal calf serum (Invitrogen GmbH) and 10 mm HEPES (Invitrogen GmbH). Cells were kept at 37 °C and 5% CO_2_ in a humidified incubator at a density of 2 × 10^5^ to 1 × 10^6^ cells mL^−1^. Cells were passaged twice a week and used from passage 9 to 17, since THP‐1 monocytes can be used up to passage 25.^[^ ^24^ ^]^

For inflammasome activation, the medium was changed and THP‐1 monocytes were left untreated or primed with 10 ng mL^−1^ lipopolysaccharide (LPS; from *Escherichia coli* 0111: B4, Sigma–Aldrich, Taufkirchen, Germany) for 4 h before 10 µm nigericin (Sigma–Aldrich) was added for further 40 min under serum‐free conditions.

### 2.5 Dosage Information

THP‐1 monocytes were incubated with only two different noncytotoxic concentrations of the BCE (15 or 50 µg mL^−1^), C3G (1 or 50 µm), FA (1 or 50 µm), CA (1 or 50 µm), or SA (1 or 50 µm) at 37 °C and 5% CO_2_ in a humidified incubator for 24 h before the NLRP3 inflammasome was activated as mentioned above. Although these concentrations were significantly lower than in other studies,^[^ ^25^, ^26^ ^]^ it must be emphasized that they do not correspond to physiological concentrations.

### 2.6 Determination of Protein Expression and ASC Speck Formation by Flow Cytometry

**Figure 1:**
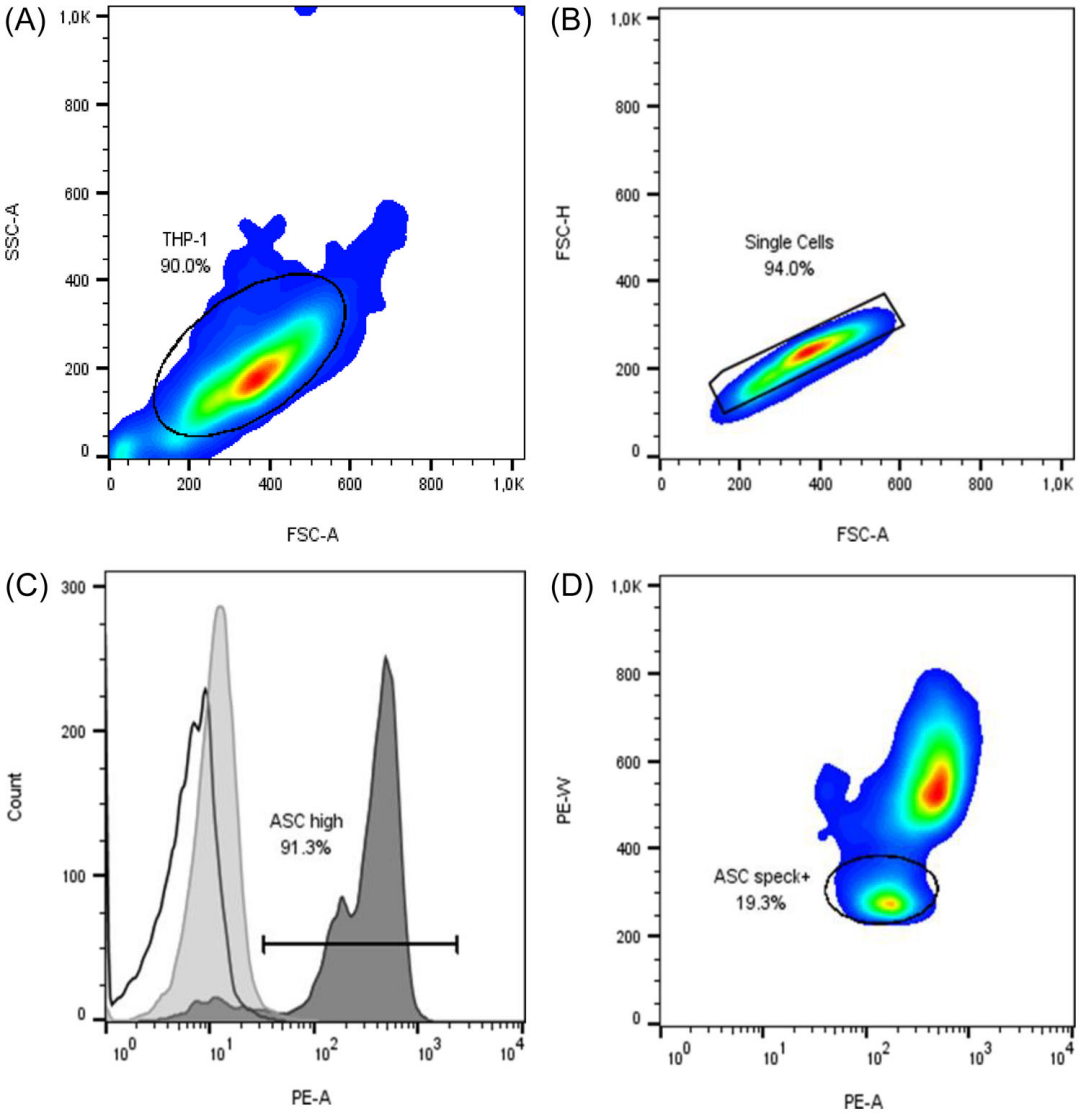
Gating strategy to assess apoptosis‐associated speck‐like protein containing a caspase recruitment domain (ASC) speck formation in THP‐1 monocytes. A) First, debris were excluded using the forward light scatter area (FSC‐A) and side scatter area (SSC‐A). B) Then, doublets were excluded using FSC‐A and FSC height (FSC‐H). C) Next, ASC positive cells (dark gray filled histogram) were selected compared to the matching isotype control (light gray filled histogram) and D) ASC speck formation was assessed via the obvious reduction in phycoerythrin width (PE‐W) due to ASC condensation. Cell density is indicated by pseudocolor plot ranging from low (blue) to high (red).

Protein expression and ASC speck formation were assessed by intracellular flow cytometry as recently published.^[^ ^23^ ^]^ In brief, cells were fixed with Cyto‐Fast Fix/Perm (BioLegend, Amsterdam, Netherlands) according to the manufacturer's instructions, and fixed cells were kept at 4 °C in azide‐containing buffer overnight. On the next day, cells were intracellularly stained with fluorescence‐labeled monoclonal antibodies against ASC (BioLegend) and NLRP3 (Miltenyi Biotec B.V. & Co. KG, Bergisch Gladbach, Germany) or the corresponding isotype controls. THP‐1 monocytes were analyzed on a BD FACSCanto II Flow Cytometer (BD Bioscience, Heidelberg, Germany) and data were analyzed with FlowJo software version 10.8.1 (BD Bioscience). ASC and NLRP3 expression were determined by comparing the median fluorescence intensity (MFI) to the corresponding isotype control and the percentage of ASC speck‐positive cells was quantified as shown in **Figure** **1**.

### 2.7 Determination of Caspase‐1 Activity

Caspase‐1 activity was measured in cultured cells using the Caspase‐Glo 1 Inflammasome Assay (Promega GmbH, Walldorf, Germany) according to the manufacturer's instructions. In brief, THP‐1 monocytes (5 × 10^4^ cells) were seeded in a white opaque 96‐well plate, and the NLRP3 inflammasome was activated by stimulating THP‐1 monocytes with LPS and/or nigericin as mentioned in section 2.4. To determine caspase‐1 activity, the Z‐WEHD aminoluciferin substrate was added and luminescence was measured after 1 h of incubation on a BioTek Synergy H1 microplate reader (BioTek GmbH, Karlsruhe, Germany).

### 2.8 Determination of Cytokine Secretion by Enzyme‐Linked Immunosorbent Assays (ELISA)

Levels of proinflammatory cytokines in cell culture supernatants were quantified using commercial IL‐1β and IL‐18 ELISA kits (Invitrogen) according to the manufacturer's instructions. Absorbance was measured at 450 nm using a BioTek Synergy H1 microplate reader (BioTek GmbH).

### 2.9 Statistical Analyses

Results are expressed as means ± standard deviation (SD) of at least three experiments and the significance level for all statistical tests was set at *p* < 0.05. Statistical calculations were performed using one‐way analysis of variance (ANOVA) followed by Dunnett's multiple comparisons test. Adjusted *p*‐values were given throughout the manuscript and all data were analyzed using GraphPad Prism version 10.0.3 (GraphPad Software, San Diego, CA, USA).

## 3 Results

### 3.1 Characterization of the Anthocyanin‐Rich Black Carrot Extract (BCE)

**Figure 2:**
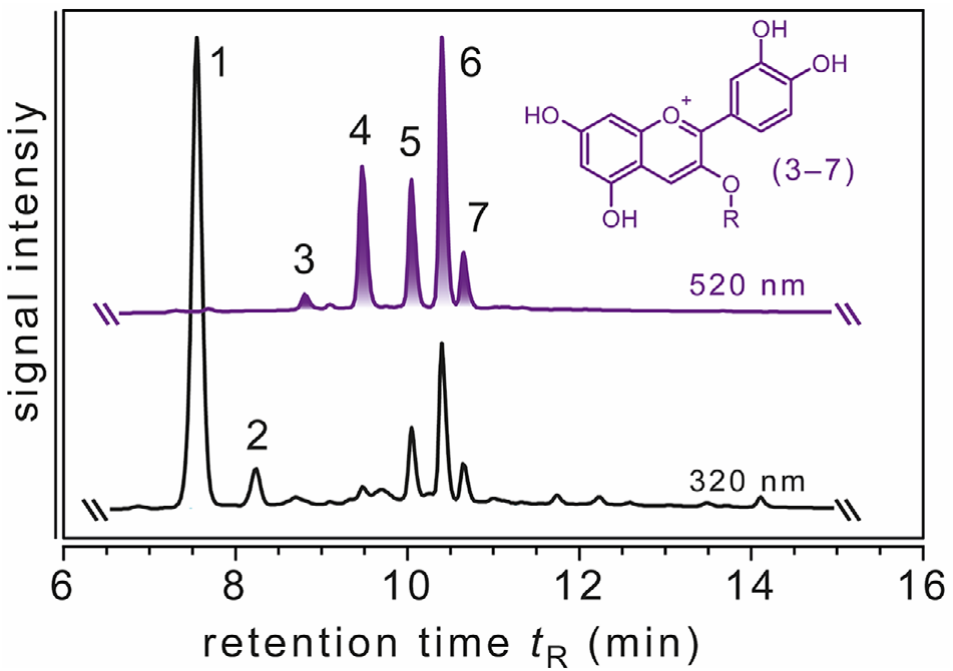
Representative HPLC‐DAD chromatograms of anthocyanins at 520 nm as well as chlorogenic and caffeic acid at 320 nm in the black carrot extract. Acylated anthocyanins also generated signals at 320 nm. Peak assignment as follows. 1) Chlorogenic acid, 2) Caffeic acid. 3) Cyanidin‐3‐xylosyl‐glucosyl‐galactoside, 4) cyanidin‐3‐xylosyl‐galactoside, 5) cyanidin‐3‐xylosyl‐(sinapoyl‐glucosyl)‐galactoside, 6) cyanidin‐3‐xylosyl‐(feruloyl‐glucosyl)‐galactoside, 7) cyanidin‐3‐xylosyl‐(*p*‐coumaroyl‐glucosyl)‐galactoside. Detailed analytical data for anthocyanin identification has been compiled in Table 1.

In agreement with previous reports on anthocyanins in black carrots,^[^ ^22^ ^]^ five different major cyanidin‐based anthocyanins as well as chlorogenic and caffeic acid were identified by HPLC‐DAD‐ESI‐QTOF‐HR‐MS/MS in the BCE (**Figure** **2**). These findings demonstrates that the extract comprised all typical black carrot anthocyanins (**Table** **1**).

**Table 1:** Identification of anthocyanins and phenolic acids in the black carrot extract by HPLC‐DAD‐ESI‐QTOF‐HR‐MS/MS.

Peak	*t*_R_ [min]	*λ*_max_ [nm]	*λ*_acyl_ [nm]	MS ^a^ ^)^ (*m*/*z*)	Sum formula	MS/MS (*m*/*z*)	Compound assignment
1	7.6	sh293, 325	–	353.0879 (353.0578)	C_16_H_17_O_9_^−^	191.0562	Chlorogenic acid (5‐caffeoylquinic acid)
2	8.2	sh291, 322	–	179.0347 (179.0350)	C_9_H_7_O_4_^−^	135.0453	Caffeic acid
3	8.8	518	–	743.2023 (743.2029)	C_32_H_39_O_20_^+^	287.0550	Cyanidin‐3‐xylosyl‐glucosyl‐galactoside
4	9.5	519	–	581.1502 (581.1501)	C_26_H_29_O_15_^+^	287.0558	Cyanidin‐3‐xylosyl‐galactoside
5	10.0	531	333	949.2608 (949.2608)	C_43_H_49_O_24_^+^	287.0554	Cyanidin‐3‐xylosyl‐(sinapoyl‐glucosyl)‐galactoside
6	10.4	530	331	919.2502 (919.2503)	C_42_H_47_O_23_^+^	287.0553	Cyanidin‐3‐xylosyl‐(feruloyl‐glucosyl)‐galactoside
7	10.7	528	318	889.2397 (889.2397)	C_41_H_45_O_22_^+^	287.0551	Cyanidin‐3‐xylosyl‐(*p*‐coumaroyl‐glucosyl)‐galactoside

*t*_R_: retention time, *λ*_max_: wavelength of maximum absorption, *λ*_acyl_: wavelength of maximum absorption in the UV range characteristic for the respective hydroxycinnamoyl moiety of acylated anthocyanins, sh: shoulder in the UV spectrum.

^a)^ MS spectra of peaks 1 and 2 displayed deprotonated molecules [M−H]^−^ in the negative, those of peaks 3–7 molecular ions [M]^+^ in the positive ion mode (theoretical *m*/*z* given in brackets).

The total anthocyanin content of the BCE was 118.1 mg g^−1^ of extract, consisting predominantly of cyanidin‐3‐xylosyl‐(feruloyl‐glucosyl)‐galactoside (47.2% w/w of total anthocyanins). Overall, acylated anthocyanins accounted for 80% w/w of the total anthocyanin content. The anthocyanin composition of the BCE is presented in **Table** **2**. Besides anthocyanins, colorless phenolic compounds like chlorogenic acid (96.7 mg g^−1^) and caffeic acid (5.7 mg g^−1^) were also present (Figure 2). The total content of phenolic compounds was 220.5 mg g^−1^.

**Table 2:** Quantification of anthocyanins and phenolic acids of the black carrot extract by HPLC‐DAD.

	[mg g^−1^]^a^ ^)^	% w/w of respective compound class
Anthocyanins^b^ ^)^		
Nonacylated		
Cyanidin‐3‐xylosyl‐glucosyl‐galactoside	3.2	2.7
Cyanidin‐3‐xylosyl‐galactoside	20.3	17.2
Acylated		
Cyanidin‐3‐xylosyl‐(sinapoyl‐glucosyl)‐galactoside	27.2	23.0
Cyanidin‐3‐xylosyl‐(feruloyl‐glucosyl)‐galactoside	55.8	47.2
Cyanidin‐3‐xylosyl‐(*p*‐coumaroyl‐glucosyl)‐galactoside	11.6	9.8
∑ Anthocyanins	118.1	100
Phenolic acids		
Chlorogenic acid^c^ ^)^	96.7	94.4
Caffeic acid^d^ ^)^	5.7	5.6
∑ Phenolic acids	102.4	100
Anthocyanin‐bound hydroxycinnamic acids^e^ ^)^		
Anthocyanin‐bound sinapinic acid	6.4	31.5
Anthocyanin‐bound ferulic acid	11.8	58.1
Anthocyanin‐bound *p*‐coumaric acid	2.1	10.3
∑ Anthocyanin‐bound hydroxycinnamic acids	20.3	100

^a)^ The black carrot extract was analyzed in duplicate by HPLC‐DAD and data are expressed as mean ± SD;

^b)^ Limit of detection (LOD) and limit of quantification (LOQ): 0.6 and 1.8 ng on column, respectively;

^c)^ LOD and LOQ: 0.5 and 1.6 ng on column, respectively;

^d)^ LOD and LOQ: 0.2 and 0.6 ng on column, respectively;

^e)^ Anthocyanin‐bound hydroxycinnamic acids were calculated from the concentration of the respective acylated anthocyanins.

### 3.2 Influence of a Black Carrot Extract (BCE) With High Amounts of Acylated Anthocyanins and Their Related Phenolic Acids on Cell Viability of THP‐1 Monocytes

After exposure to increasing concentrations of the BCE, C3G or phenolic acids for 24 h cell viability of THP‐1 monocytes was assessed by flow cytometry to determine potential cytotoxic effects. Cell viability was only slightly affected (from 95.5% ± 1.0% to 92.12% ± 1.9%) by preincubation with the highest concentration of SA (*p* < 0.01) (Supporting Information Figure S1). Therefore, noncytotoxic high and low concentrations were selected to examine the inhibitory effects of the BCE, C3G, or phenolic acids on NLRP3 inflammasome activation.

### 3.3 Influence of a Black Carrot Extract (BCE) With High Amounts of Anthocyanins and Their Related Phenolic Acids on ASC and NLRP3 Protein Expression in THP‐1 Monocytes

**Figure 3:**
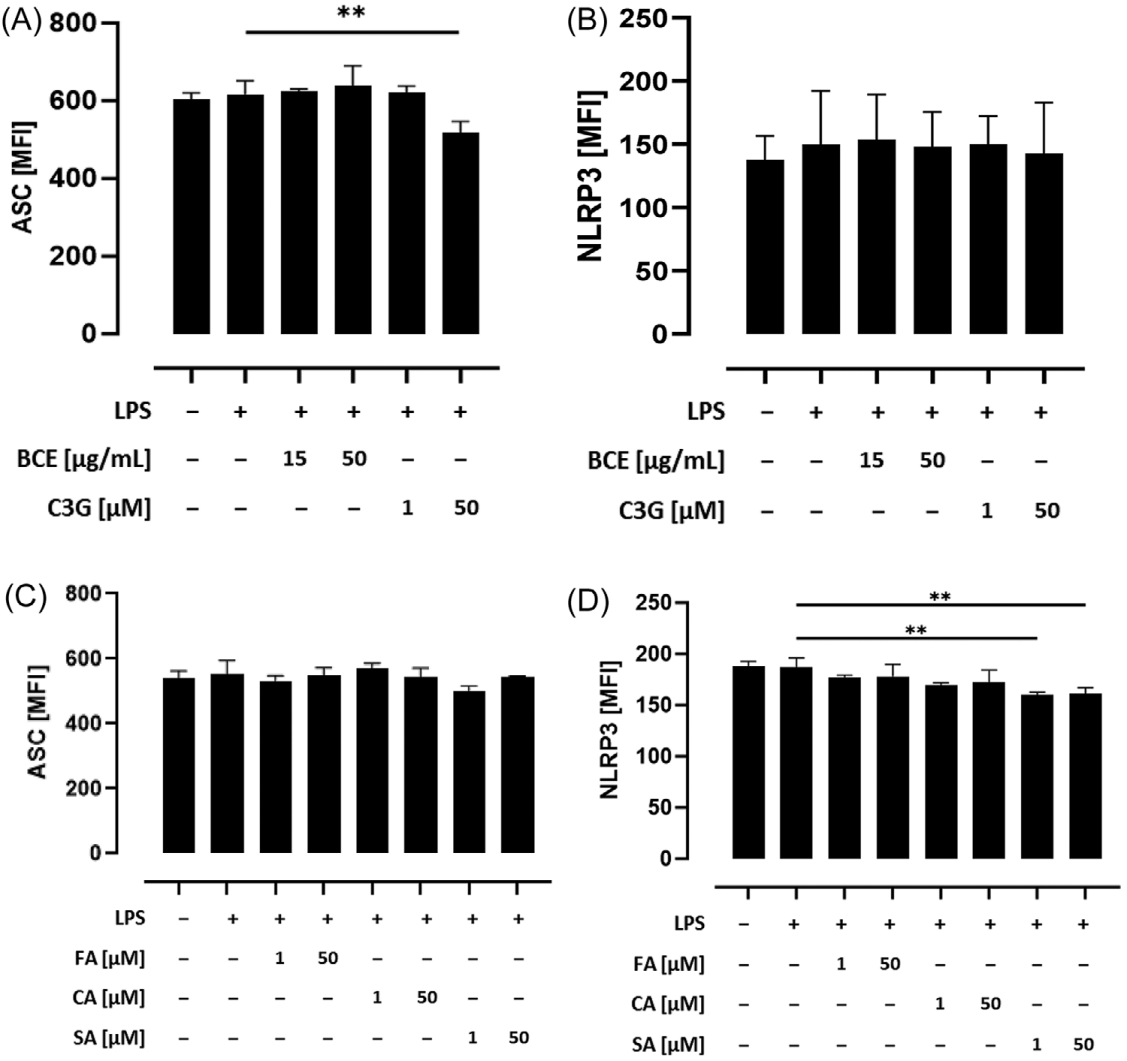
Influence of a black carrot extract (BCE) with high amounts of acylated anthocyanins and their related phenolic acids on ASC and NLRP3 protein expression in THP‐1 monocytes. THP‐1 monocytes were preincubated with the indicated concentrations of the BCE, C3G, or phenolic acids before cells were primed with LPS. A, C) ASC and B, D) NLRP3 protein expression were assessed as median fluorescence intensity (MFI) by intracellular flow cytometry. Significant differences to LPS primed cells were calculated using one‐way ANOVA with Dunnett's multiple comparisons test (**p < 0.01). ANOVA, analysis of variance; ASC, apoptosis‐associated speck‐like protein containing a caspase recruitment domain; cyanidin‐3‐glucoside (C3G); *p*‐coumaric acid (CA); ferulic acid (FA); LPS, lipopolysaccharide; NLRP3, nucleotide‐binding oligomerization domain‐like receptor pyrin domain‐containing protein 3; sinapinic acid (SA).

Priming of the NLRP3 inflammasome by LPS, which is a component of the outer membrane of Gram‐negative bacteria such as *E. coli*,^[^ ^1^ ^]^ is a common method to induce transcriptional upregulation of the NLRP3 inflammasome.^[^ ^5^ ^]^ To determine potential mechanisms by which anthocyanins and their related phenolic acids attenuate NLRP3 inflammasome activation, THP‐1 monocytes were left untreated or primed with LPS before ASC, as well as NLRP3, protein expression was assessed by intracellular flow cytometry. As shown in **Figure** **3**A, pretreatment with the high C3G concentration significantly declined ASC protein expression compared to the LPS primed control (*p* < 0.01), while no effect of C3G and the BCE on NLRP3 protein expression was observed (Figure 3B). In contrast, incubation of cells with phenolic acids prior to LPS priming was uneffective on ASC protein expression (Figure 3C), and NLRP3 protein expression of LPS primed cells was decreased only by preincubation with low and high concentrations of SA (*p* < 0.01; Figure 3D). Taken together, these results suggest that C3G and phenolic acids may diminish NLRP3 inflammasome priming by attenuating ASC and NLRP3 protein expression.

### 3.4 Influence of a Black Carrot Extract (BCE) With High Amounts of Anthocyanins and Their Related Phenolic Acids on ASC Speck Formation in THP‐1 Monocytes

**Figure 4:**
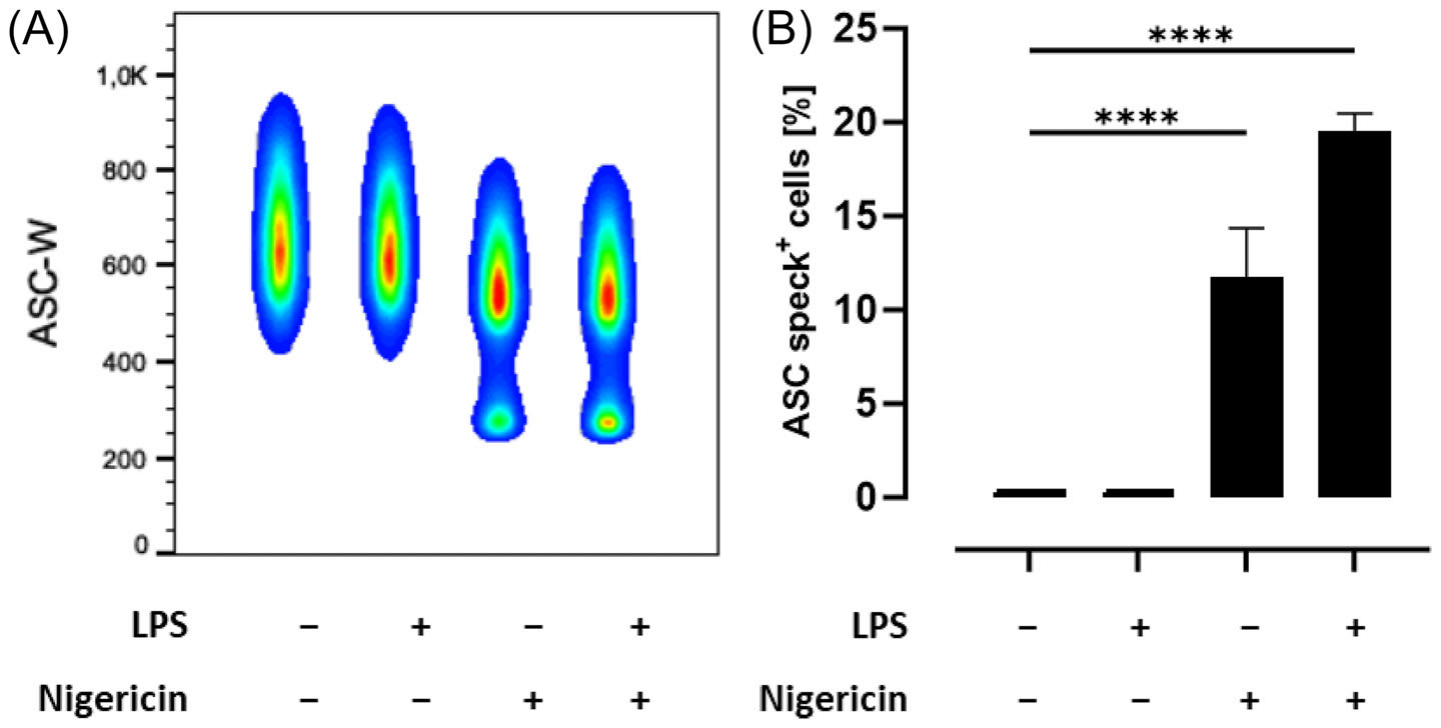
ASC speck formation in THP‐1 monocytes. A) ASC pulse width (ASC‐W) analysis of THP‐1 monocytes by flow cytometry. Cells were treated with LPS and/or nigericin as indicated. Unstimulated cells were used as a negative control. A representative data set (*n* = 1) with 14.000 cells per treatment is shown and cell density is indicated by a pseudocolor plot ranging from low (blue) to high (red). B) The percentage of ASC speck‐positive cells was quantified. Significant differences to untreated cells were calculated using one‐way ANOVA with Dunnett's multiple comparisons test (****p < 0.0001). ANOVA, analysis of variance; ASC, apoptosis‐associated speck‐like protein containing a caspase recruitment domain; lipopolysaccharide (LPS).

Although priming of the NLRP3 inflammasome facilitates inflammasome assembly, a second signal is needed to activate the NLRP3 inflammasome. This second step can be mediated by a broad range of inflammasome inducers such as nigericin, resulting in the oligomerization of NLRP3, ASC, and pro‐caspase‐1 to a supramolecular ASC speck.^[^ ^27^ ^]^ Sester et al. recently established a new method to assess NLRP3 inflammasome assembly by intracellular flow cytometry.^[^ ^28^ ^]^ Upon NLRP3 inflammasome formation, ASC proteins condensate, which could be detected by decreased ASC pulse width as shown in **Figure** **4**A. Therefore, THP‐1 monocytes were left untreated or primed with LPS for several hours followed by activation of the NLRP3 inflammasome with nigericin as the second stimulus. Cells with decreased width, detected by intracellular flow cytometry, were considered ASC speck‐positive, and the percentage of ASC speck‐positive cells was quantified. As expected, ASC speck formation significantly increased after stimulation with LPS and nigericin compared to untreated cells (*p* < 0.0001), although nigericin treatment alone was also sufficient to induce ASC speck formation in THP‐1 monocytes (*p* < 0.0001). In contrast, we could not observe any spontaneous ASC speck formation in untreated and LPS‐treated THP‐1 monocytes (Figure 4B).

**Figure 5:**
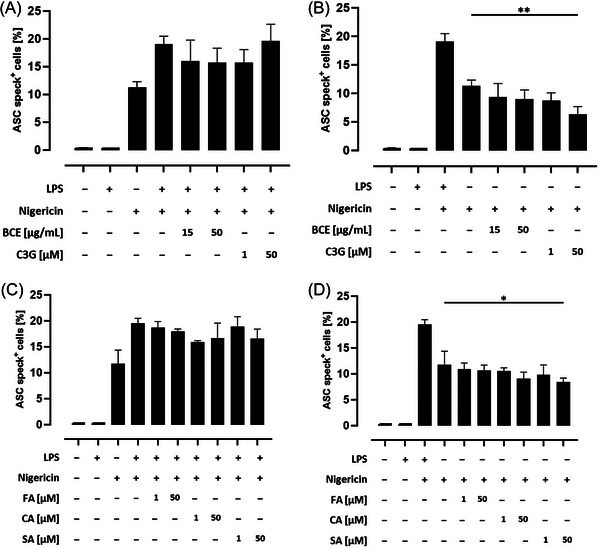
Influence of a black carrot extract (BCE) with high amounts of acylated anthocyanins and their related phenolic acids on ASC speck formation in THP‐1 monocytes. THP‐1 monocytes were preincubated with the indicated concentrations of the BCE, cyanidin‐3‐glucoside, or phenolic acids before the NLRP3 inflammasome was activated. Significant differences to A, C) LPS and nigericin stimulated cells or B, D) only nigericin treated cells were calculated using one‐way ANOVA with Dunnett's multiple comparisons test (*p < 0.05 and **p < 0.01). ANOVA, analysis of variance; ASC, apoptosis‐associated speck‐like protein containing a caspase recruitment domain; C3G; CA; FA; LPS, lipopolysaccharide; NLRP3, nucleotide‐binding oligomerization domain‐like receptor pyrin domain‐containing protein 3; SA.

We next investigated the influence of the BCE with high amounts of acylated anthocyanins and their related phenolic acids on ASC speck formation, which was slightly but not significantly declined by pretreatment with the BCE and the lower concentration of C3G (**Figure** **5**A). In contrast, pretreatment with both concentrations of C3G reduced ASC speck formation in unprimed, nigericin‐stimulated cells, whereas the effect of the higher concentration was still significant after correction for multiple testing (*p* < 0.01; Figure 5B). In addition, pretreatment with both concentrations of CA and the higher dose of SA declined ASC speck formation in LPS and nigericin‐treated cells. However, after correction for multiple comparisons, these effects were no longer statistically significant (Figure 5C). In contrast, ASC speck formation decreased by pretreatment with high concentrations of CA and SA prior to inflammasome activation in unprimed THP‐1 monocytes, whereas the inhibitory effect of SA was also statistically significant after correction for multiple testing (*p* < 0.05; Figure 5D). In conclusion, these results indicate that preincubation of THP‐1 monocytes with the related phenolic acid such as SA may attenuate ASC speck formation, while inhibitory effects are greater in unprimed cells.

### 3.5 Influence of a Black Carrot Extract (BCE) With High Amounts of Acylated Anthocyanins and Their Related Phenolic Acids on Caspase‐1 Activity in THP‐1 Monocytes

**Figure 6:**
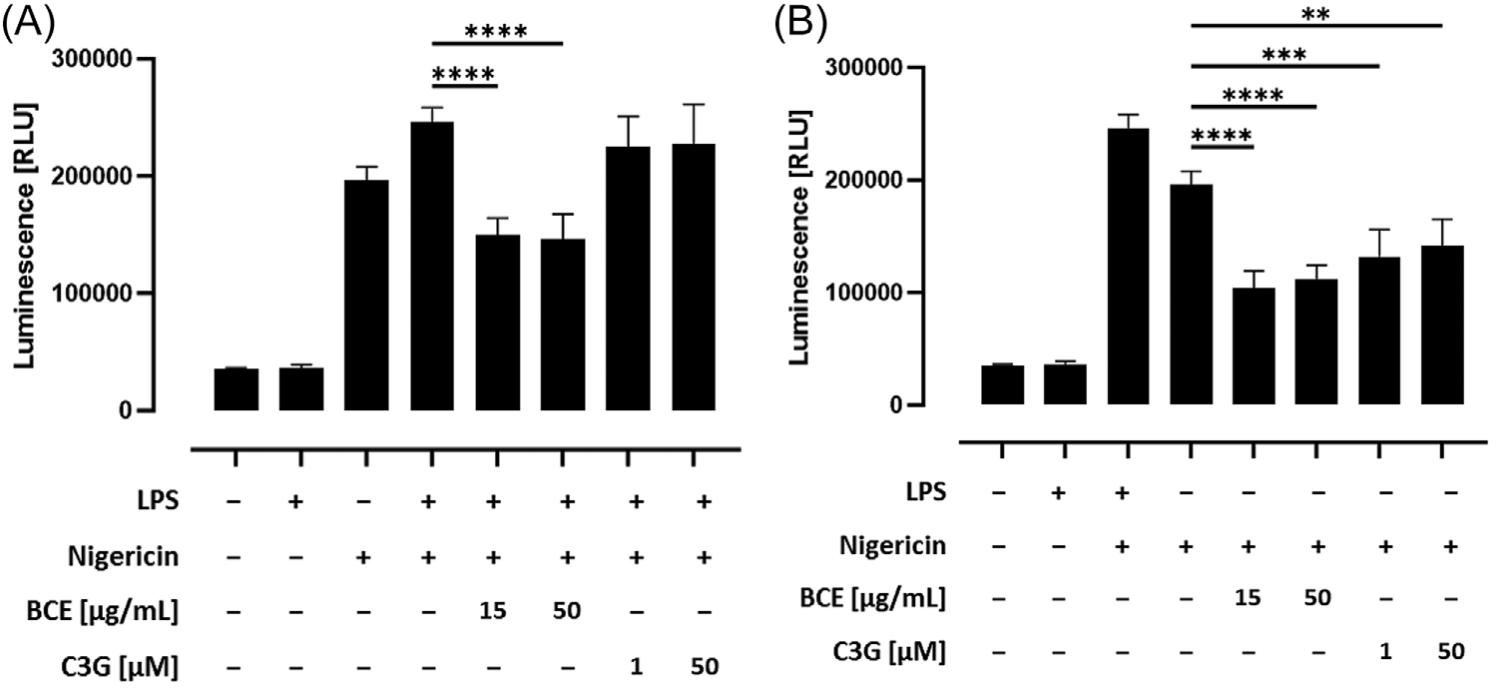
Influence of a black carrot extract (BCE) with high amounts of acylated anthocyanins on caspase‐1 activity in THP‐1 monocytes. THP‐1 monocytes were preincubated with the indicated concentrations of the BCE or C3G before the NLRP3 inflammasome was activated. Caspase‐1 activity was measured by using a bioluminescent assay and luminescence was measured as relative light unit (RLU). Significant differences to (A) LPS and nigericin stimulated cells or (B) only nigericin treated cells were calculated using one‐way ANOVA with Dunnett's multiple comparisons test (**p < 0.01, ***p < 0.001, and ****p < 0.0001). ANOVA, analysis of variance; C3G, cyanidin‐3‐glucoside; LPS, lipopolysaccharide; NLRP3, nucleotide‐binding oligomerization domain‐like receptor pyrin domain‐containing protein 3.

ASC speck formation leads to the activation of caspase‐1. Hence, we next examined the influence of the BCE and their related phenolic acids on caspase‐1 activity in THP‐1 monocytes. A bioluminescent assay was used to selectively measure caspase‐1 activity,^[^ ^29^ ^]^ which was significantly increased in LPS primed and nigericin‐activated THP‐1 monocytes (*p* < 0.0001; **Figure** **6**A) compared to untreated THP‐1 monocytes. To a lower extent, caspase‐1 activity also increased in unprimed nigericin‐activated THP‐1 monocytes (*p* < 0.0001; Figure 6A). However, preincubation with the BCE resulted in a significant decline of caspase‐1 activity, both in LPS primed and nigericin‐activated (*p* < 0.0001; Figure 6A) as well as in unprimed nigericin‐activated cells (*p* < 0.0001; Figure 6B). In addition, caspase‐1 activity also significantly declined in unprimed nigericin‐activated cells (Figure 6B) after preincubation with both concentrations of C3G (*p* < 0.001 and *p* < 0.05, respectively). In contrast, caspase‐1 activity was not affected by C3G in LPS‐primed and nigericin‐activated cells (Figure 6A). Interestingly, caspase‐1 activity was also not declined after inflammasome activation by pretreatment with phenolic acids (Supporting Information Figure S2). Consequently, these findings suggest that a BCE with high amounts of acylated anthocyanins and C3G reduce caspase‐1 activity in THP‐1 monocytes.

### 3.6 Influence of a Black Carrot Extract (BCE) With High Amounts of Acylated Anthocyanins and Their Related Phenolic Acids on Proinflammatory Cytokine Release in THP‐1 Monocytes

**Figure 7:**
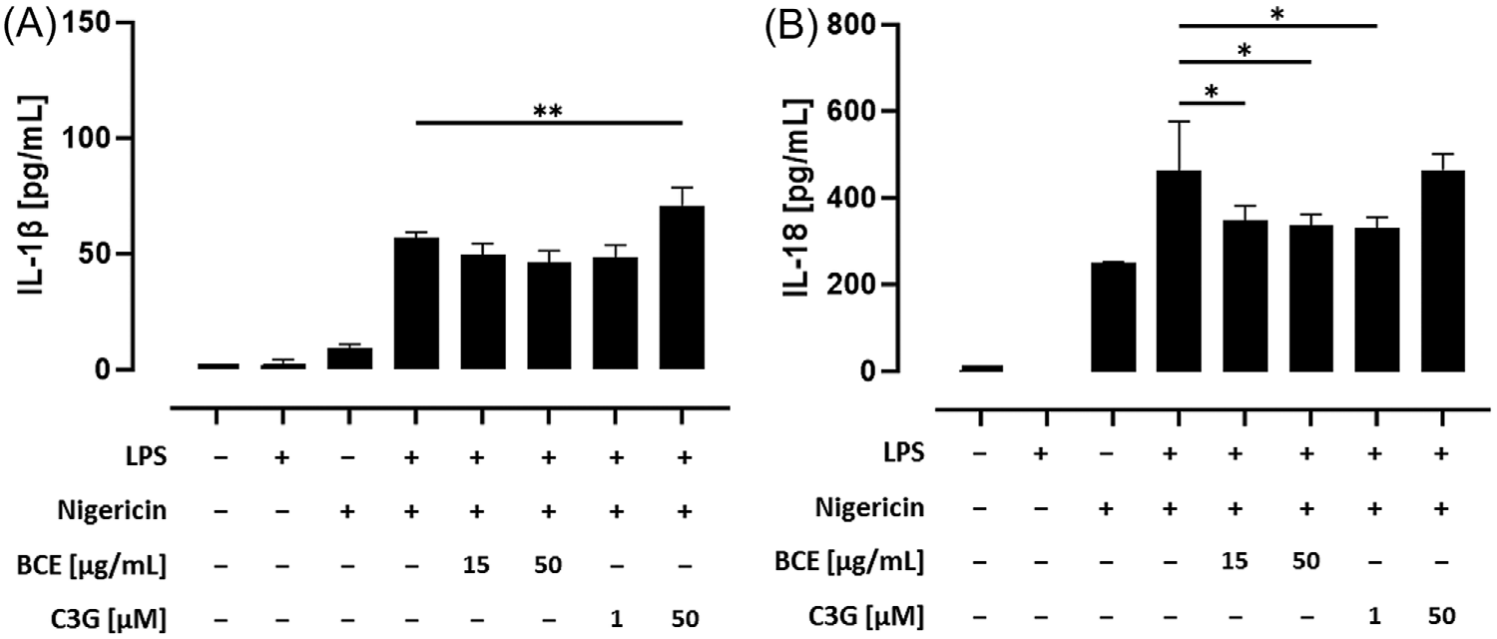
Influence of a black carrot extract (BCE) with high amounts of acylated anthocyanins on proinflammatory cytokine release in THP‐1 monocytes. THP‐1 monocytes were preincubated with the indicated concentrations of BCE or C3G before the NLRP3 inflammasome was activated. Release of (A) IL‐1β and (B) IL‐18 into the cell culture supernatant was measured by ELISA. Significant differences to LPS and nigericin‐stimulated cells were calculated using one‐way ANOVA with Dunnett's multiple comparisons test (*p < 0.05 and **p < 0.01). ANOVA, analysis of variance; C3G, cyanidin‐3‐glucoside; CA, *p*‐coumaric acid; ELISA, enzyme‐linked immunosorbent assays; FA, ferulic acid; LPS, lipopolysaccharide; NLRP3, nucleotide‐binding oligomerization domain‐like receptor pyrin domain‐containing protein 3; SA, sinapinic acid.

IL‐1β and IL‐18 are inflammatory cytokines which belong to the IL‐1 superfamily.^[^ ^6^ ^]^ Active caspase‐1 catalyzes the maturation of their proforms into their mature forms, which are subsequent released.^[^ ^5^ ^]^ To measure the influence of a BCE with high amounts of acylated anthocyanins and their related phenolic acids on proinflammatory cytokine release, IL‐1β and IL‐18 concentrations in cell culture supernatants were measured by ELISA. IL‐1β secretion was significantly induced by nigericin treatment (*p* < 0.05) compared to the untreated control. However, prior priming of THP‐1 monocytes with LPS lead to a six‐fold increase of IL‐1β secretion compared to nigericin‐treated cells. Similarly, IL‐18 was also significantly increased in the supernatant of nigericin, as well as LPS and nigericin, treated cells compared to the untreated control (*p *< 0.05 and *p *< 0.0001, respectively). Pretreatment with the high concentration of the BCE (50 µg mL^−1^) and the low concentration of C3G (1 µm) resulted in a reduced IL‐1β release. However, after correction for multiple testing, the observed inhibitory effects were no longer statistically significant. Surprisingly, IL‐1β secretion was significantly increased by pretreatment with the high C3G concentration (*p* < 0.01; **Figure** **7**A). In addition, IL‐18 release into the cell culture supernatant was significantly decreased by both concentrations of the BCE (*p* < 0.05). Furthermore, IL‐18 was also declined by pretreatment with the lower concentration of C3G (*p* < 0.05), while the higher concentration had no effect (Figure 7B). In contrast, neither IL‐1β nor IL‐18 concentrations were significantly declined by pretreatment with phenolic acids (Supporting Information Figure S3).

Taken together, our results indicate that a BCE with high amounts of acylated anthocyanins as well as low concentrations of C3G diminish proinflammatory IL‐18 cytokine release in THP‐1 monocytes. It was also observed that incubation of THP‐1 cells with C3G at high concentrations resulted in a significantly increased release of IL‐1β, which was an unexpected outcome.

## 4 Discussion

Overweight and obesity promote chronic low‐grade inflammation, which is characterized by increased circulating proinflammatory cytokine levels.^[^ ^30^, ^31^ ^]^ Obesity‐induced metabolic stress favors excessive activation of the NLRP3 inflammasome resulting in proinflammatory cytokine release,^[^ ^32^ ^]^ which seems to be one underlying mechanism via that circulating monocytes contribute to systemic low‐grade inflammation. Consequently, food components targeting NLRP3 inflammasome activation may be a novel safe and effective strategy to prevent the initiation and progression of inflammation‐related diseases. Therefore, the present study aimed to determine the influence of a BCE with high amounts of acylated anthocyanins and their related phenolic acids (i.e., FA,, CA, and SA) as well as C3G as a non‐acylated anthocyanin on multiple steps of the NLRP3 inflammasome activation cascade in THP‐1 monocytes.

Canonical NLRP3 inflammasome activation usually comprises two steps. The first step (priming) can be mediated by different damage‐associated molecular patterns (DAMPs) and pathogen‐associated molecular patterns (PAMPs) which bind to pattern recognition receptors (PRRs) such as the Toll‐like receptors (TLRs), nucleotide‐binding oligomerization domain 2 (NOD2), and receptors for advanced glycation end products (RAGE).^[^ ^32^ ^]^ Binding by different agonists (e.g., LPS) results in the activation of several signaling pathways. In this context, binding of LPS to TLR4 activated the nuclear factor kappa B (NFκB) signaling pathway, which in turn induces de novo synthesis of NLRP3 inflammasome components.^[^ ^32^ ^]^ Hence, inhibition of, e.g., TLR4/NFκB signaling pathway may effectively diminish NLRP3 inflammasome priming. Our results show that C3G and phenolic acids attenuate ASC and NLRP3 protein expression in THP‐1 monocytes. Similarly, C3G supplementation reduced ASC and NLRP3 expression in the liver tissue of ethanol‐induced steatohepatitis mice by NFκB suppression.^[^ ^33^ ^]^ Administration of FA and CA also reduced ASC, as well as NLRP3, protein expression in other rodent tissues.^[^ ^34^, ^35^ ^]^ In addition, FA decreased LPS‐induced NLRP3 expression in THP‐1‐derived macrophages.^[^ ^36^ ^]^ Furthermore, CA treatment attenuated high‐fat and high‐sucrose diet‐induced hepatic TLR4 and NFκB target gene expression in mice.^[^ ^37^ ^]^ Taken together these evidence suggest that anthocyanins and phenolic acids may mitigate NLRP3 and ASC expression in human monocytes through downregulation of the TLR4/NFκB signaling pathway.

Although priming of the NLRP3 inflammasome induces the transcriptional upregulation of NLRP3 inflammasome components, NLRP3 inflammasome assembly is only induced by a second independent signal. However, in contrast to murine models and human macrophages, NLRP3 activation can also take place in unprimed human monocytes.^[^ ^27^, ^38^ ^]^

NLRP3 inflammasome activation is mediated by a broad range of metabolic and environmental inflammasome inducers, resulting in the oligomerization of NLRP3, ASC, and pro‐caspase‐1 to a supramolecular ASC speck.^[^ ^27^ ^]^ Our results suggest that preincubation of THP‐1 monocytes with BCE with high amounts of acylated anthocyanins and their related phenolic acids attenuate ASC speck formation in human monocytes, whereas the observed inhibitory effects are greater in unprimed cells. Similarly, pretreatment with SA diminished ASC speck formation in bone marrow‐derived macrophages.^[^ ^39^ ^]^ Interestingly, in silico analyses indicate that phenolic acids can bind to NLRP3 and ASC molecules.^[^ ^34^, ^40^ ^]^ Thus, phenolic acids potentially reduce NLRP3 inflammasome activation by preventing intermolecular interactions of NLRP3 components.^[^ ^40^ ^]^ Maybe anthocyanins are also able to form hydrogen bonds to active site amino acids of NLRP3 components by their A‐ and B‐ring hydroxyl groups. This could explain the observed inhibitory effects of C3G on ASC speck formation in the present study. Furthermore, it is well established that oxidative stress induces NLRP3 inflammasome activation, and several studies have shown that anthocyanins and phenolic acids attenuate oxidative stress and prevent reactive oxygen species generation.^[^ ^33^, ^35^, ^36^, ^41^ ^]^ Hence, BCE with high amounts of acylated anthocyanins and their related phenolic acids could also diminish ASC speck formation by downregulation of the activation signal.

Oligomerization of the NLRP3 inflammasome results in the autoproteolytic activation of caspase‐1, which in turn catalyzes the maturation and release of IL‐1β and IL‐18.^[^ ^5^, ^6^ ^]^ These inflammatory cytokines can activate other immune cells and thus further increase the secretion of inflammatory cytokines.^[^ ^42^ ^]^ Furthermore, active caspase‐1 also mediates an inflammatory form of programmed cell death called pyroptosis through gasdermin‐D cleavage.^[^ ^5^ ^]^ The N‐terminal domain of cleaved gasdermin D oligomerizes and forms pores into the cell membrane. The loss of cell integrity leads to the release of other alarmins apart from cytokines, which further promote the inflammatory response.^[^ ^32^ ^]^ Therefore, inhibition of caspase‐1 activity and subsequent proinflammatory cytokine release may be effective to reduce low‐grade inflammation. Our results show that the BCE and C3G decline caspase‐1 activity in THP‐1 monocytes. Inhibition of caspase‐1 activity was induced in LPS‐primed and unprimed cells by the BCE (15 and 50 µg mL^−1^) and only in unprimed cells by C3G (1 µm). In the case of the BCE, this inhibition was not dose dependent, suggesting that lower concentrations may also be able to induce an effect. However, the inhibition was only associated with a release of IL‐18 but not IL‐1β in LPS‐primed THP‐1 monocytes by the BCE. Using a low concentration of C3G (1 µm), we only observed a reduced release of IL‐18 in LPS‐primed cells, whereas higher concentrations (50 µm) were ineffective, but surprisingly induced IL‐1β release from LPS‐primed cells. In mice with alcoholic steatohepatitis, dietary C3G suppressed hepatic expression of active caspase‐1, as well as IL‐1β and IL‐18 expression levels.^[^ ^33^ ^]^ Similarly, oral gavage of “purple sweet potato color”, which main components were acylated cyanidin and peonidin glycosides with acyl moieties such as FA (‐feruloyl), CA (‐coumaroyl), and caffeic acid (‐caffeoyl), reduced protein levels of cleaved caspase‐1 and IL‐1β in high‐fat diet‐treated mice livers and kidneys.^[^ ^41^, ^43^ ^]^ In addition, several cell and animal models have shown that phenolic acids such as FA, CA, and SA reduce transcriptional, as well as protein expression, levels of caspase‐1 and IL‐1β.^[^ ^34^, ^35^, ^36^, ^37^, ^39^ ^]^ In contrast to these findings, neither caspase‐1 activity nor inflammatory cytokine release is diminished by phenolic acids in the present study. However, discrepancies in study results may be due to experimental and methodological differences, since very high phenolic acid concentrations up to 200 µm were used in some studies.^[^ ^37^, ^39^ ^]^

Taken together, our data suggest for the first time that a BCE with high amounts of acylated anthocyanins and their related phenolic acids diminish priming and activation of the NLRP3 inflammasome in THP‐1 monocytes. Certainly, our study may have some limitations. First, we used THP‐1 cells. Monocytic cell lines, such as THP‐1 cells, are typically derived from patients with neoplasms, thus their responses may deviate markedly from blood‐derived monocytes. Depending on the investigated endpoint, the values can be many times lower or higher than in noncarcinogenic cells isolated from blood.^[^ ^44^, ^45^ ^]^ However, THP‐1 cells are a common cell model to study NLRP3 inflammasome activation^[^ ^5^ ^]^ and priming is disposable in THP‐1 monocytes similar to primary human monocytes.^[^ ^27^, ^37^ ^]^ Secondly, dietary phenolic acids have mainly been detected as glucuronides, sulfates, or methylated metabolites in vivo.^[^ ^46^ ^]^ However, those Phase II metabolites were not tested in our in vitro models. Third, only two different concentrations were used for both the BCE and phenolic acids. Since no concentration‐dependent effects were observed for some parameters, significantly lower concentrations could also have effects, which should be verified in further studies.

To the best of our knowledge, this is the first study investigating the influence of a BCE with high amounts of acylated anthocyanins and their related phenolic acids on NLRP3 inflammasome activation in human THP‐1 monocytes. Our results show that the BCE and the aforementioned phenolic acids, which could be a microbial fermentation product of the acylated anthocyanins from the BCE in the colon, could be responsible for diminished priming and activation of the NLRP3 inflammasome in THP‐1 monocytes. Although phenolic acids can be formed as a microbial end product in the gastrointestinal tract, the BCE has a variety of other phenolic compounds, including chlorogenic acid, caffeic acid, and nonacylated anthocyanins. These compounds could also be responsible for the observed effects. In view of the previous results, further studies with corresponding metabolites or isolated acylated anthocyanins should be carried out to confirm the present findings with THP‐1 monocytes. In addition, the usage of primary human monocytes is necessary to provide conclusions that are even more definitive. Additionally, investigating intracellular targets of the signal cascade such as TLR4/NF‐kB should also been included as this signaling cascade play a huge role in NLRP3 inflammasome activation.

## Conflict of Interest

The authors declare no conflict of interest.

## Author Contributions

I.B.: Conceptualization, methodology, formal analysis, investigation, writing—original draft, Visualization. K.B.: Analysis and characterization of the black carrot extract. C.S.: Analysis and characterization of the black carrot extract. R.S.: Writing—review & editing. G.M.: Methodology, writing—review & editing. E.F., D.G., Z.M., and H.P.D.: Investigation. M.F.: Writing—review & editing. M.S.: Methodology, Writing—review & editing, Visualization. S.K.: Conceptualization, methodology, supervision, writing—review & editing.

## Supporting information



Supporting Information

## Data Availability

Data is available on request from the authors.

## References

[1] D. Zheng , T. Liwinski , E. Elinav , Cell Discov 2020, 6, 36. 10.1038/s41421-020-0167-x PMC7280307 32550001

[2] C. Vetrani , A. Di Nisio , S. A. Paschou , L. Barrea , G. Muscogiuri , C. Graziadio , S. Savastano , A. Colao , Nutrients 2022, 14, 2103. 35631244 10.3390/nu14102103 PMC9145366

[3] A. Zahid , B. Li , A. J. K. Kombe , T. Jin , J. Tao , Front. Immunol. 2019, 10, 2538. 31749805 10.3389/fimmu.2019.02538 PMC6842943

[4] F. Hoss , J. F. Rodriguez‐Alcazar , E. Latz , Cell. Mol. Life Sci. 2017, 74, 1211. 27761594 10.1007/s00018-016-2396-6 PMC11107573

[5] G. Zito , M. Buscetta , M. Cimino , P. Dino , F. Bucchieri , C. Cipollina , Int. J. Mol. Sci. 2020, 21, 4294. 32560261 10.3390/ijms21124294 PMC7352206

[6] J. C. Ralston , C. L. Lyons , E. B. Kennedy , A. M. Kirwan , H. M. Roche , Annu. Rev. Nutr. 2017, 37, 77. 28826373 10.1146/annurev-nutr-071816-064836

[7] C. Ngamsamer , J. Sirivarasai , N. Sutjarit , Biomolecules 2022, 12, 852. 35740977 10.3390/biom12060852 PMC9230453

[8] J. Yañez‐Apam , A. Domínguez‐Uscanga , A. Herrera‐González , J. Contreras , L. Mojica , G. Mahady , D. A. Luna‐Vital , Pharmaceuticals (Basel) 2023, 16, 638. 37242421 10.3390/ph16050638 PMC10220857

[9] I. Krga , D. Milenkovic , J. Agric. Food Chem. 2019, 67, 1771. 30698008 10.1021/acs.jafc.8b06737

[10] E. C. Montilla , M. R. Arzaba , S. Hillebrand , P. Winterhalter , J. Agric. Food Chem. 2011, 59, 3385. 21381748 10.1021/jf104724k

[11] J. Jokioja , B. Yang , K. M. Linderborg , Compr. Rev. Food Sci. Food Saf. 2021, 20, 5570. 34611984 10.1111/1541-4337.12836

[12] X. Wu , G. R. Beecher , J. M. Holden , D. B. Haytowitz , S. E. Gebhardt , R. L. Prior , J. Agric. Food Chem. 2006, 54, 4069. 16719536 10.1021/jf060300l

[13] G. Mazza , C. D. Kay , T. Cottrell , B. J. Holub , J. Agric. Food Chem. 2002, 50, 7731. 12475297 10.1021/jf020690l

[14] J. Prada‐Muñoz , E. Coy‐Barrera , Molecules 2024, 29, 691. 38338435 10.3390/molecules29030691 PMC10855998

[15] A. C. Kurilich , B. A. Clevidence , S. J. Britz , P. W. Simon , J. A. Novotny , J. Agric. Food Chem. 2005, 53, 6537. 16076146 10.1021/jf050570o

[16] M. Iorizzo , J. Curaba , M. Pottorff , M. G. Ferruzzi , P. Simon , P. F. Cavagnaro , Genes (Basel) 2020, 11, 906. 32784714 10.3390/genes11080906 PMC7465225

[17] K. Chen , M. K. Kortesniemi , K. M. Linderborg , B. Yang , J. Agric. Food Chem. 2023, 71, 1002. 36515085 10.1021/acs.jafc.2c05879 PMC9853865

[18] C. S. Charron , A. C. Kurilich , B. A. Clevidence , P. W. Simon , D. J. Harrison , S. J. Britz , D. J. Baer , J. A. Novotny , J. Agric. Food Chem. 2009, 57, 1226. 19166298 10.1021/jf802988s

[19] J. Fleschhut , F. Kratzer , G. Rechkemmer , S. E. Kulling , Eur. J. Nutr. 2006, 47, 7. 10.1007/s00394-005-0557-8 15834757

[20] J. Jokioja , J. Percival , M. Philo , B. Yang , P. A. Kroon , K. M. Linderborg , Mol. Nutr. Food Res. 2021, 65, e2000898. 33687145 10.1002/mnfr.202000898

[21] C. D. Kay , Nutr. Res. Rev. 2006, 19, 137. 19079881 10.1079/NRR2005116

[22] C. C. Gras , R. Carle , R. M. Schweiggert , J. Food Compos. Anal. 2015, 44, 170.

[23] I. Behrendt , I. Röder , F. Will , G. Michel , E. Friedrich , D. Grote , Z. Martin , H. P. Dötzer , M. Fasshauer , M. Speckmann , S. Kuntz , Metabolites 2024, 14, 203. 38668331 10.3390/metabo14040203 PMC11051782

[24] W. Chanput , J. J. Mes , H. J. Wichers , Int. Immunopharmacol. 2014, 23, 37. 25130606 10.1016/j.intimp.2014.08.002

[25] M. Zaim , I. Kara , A. Muduroglu , Cytotechnology 2021, 73, 827. 34776632 10.1007/s10616-021-00500-4 PMC8554904

[26] C. Sevimli‐Gur , B. Cetin , S. Akay , S. Gulce‐Iz , O. Yesil‐Celiktas , Plant Foods Hum. Nutr. 2013, 68, 293. 23828497 10.1007/s11130-013-0371-z

[27] A. Gritsenko , S. Yu , F. Martin‐Sanchez , I. Diaz‐Del‐Olmo , E.‐M. Nichols , D. M. Davis , D. Brough , G. Lopez‐Castejon , Front. Immunol. 2020, 11, 565924. 33101286 10.3389/fimmu.2020.565924 PMC7555430

[28] D. P. Sester , S. J. Thygesen , V. Sagulenko , P. R. Vajjhala , J. A. Cridland , N. Vitak , K. W. Chen , G. W. Osborne , K. Schroder , K. J. Stacey , J. Immunol. 2015, 194, 455. 25404358 10.4049/jimmunol.1401110

[29] M. O'Brien , D. Moehring , R. Muñoz‐Planillo , G. Núñez , J. Callaway , J. Ting , M. Scurria , T. Ugo , L. Bernad , J. Cali , D. Lazar , J. Immunol. Methods 2017, 447, 1. 28268194 10.1016/j.jim.2017.03.004

[30] R. T. Mattos , N. I. Medeiros , C. A. Menezes , R. C. G. Fares , E. P. Franco , W. O. Dutra , F. Rios‐Santos , R. Correa‐Oliveira , J. A. S. Gomes , PLoS ONE 2016, 11, e0168610. 27977792 10.1371/journal.pone.0168610 PMC5158089

[31] M. M. J. van Greevenbroek , C. G. Schalkwijk , C. D. A. Stehouwer , Neth. J. Med. 2013, 71, 174. 23723111

[32] M. Groslambert , B. F. Py , J. Inflamm. Res. 2018, 11, 359. 30288079 10.2147/JIR.S141220 PMC6161739

[33] Y. Zhou , S. Wang , T. Wan , Y. Huang , N. Pang , X. Jiang , Y. Gu , Z. Zhang , J. Luo , L. Yang , Free Radic. Biol. Med. 2020, 160, 334. 32805401 10.1016/j.freeradbiomed.2020.08.006

[34] H. M. Doss , C. Dey , C. Sudandiradoss , M. K. Rasool , Life Sci. 2016, 148, 201. 26851531 10.1016/j.lfs.2016.02.004

[35] M. Kinra , N. Ranadive , M. Nampoothiri , D. Arora , J. Mudgal , Naunyn Schmiedebergs Arch. Pharmacol. 2024, 397, 1829. 37755515 10.1007/s00210-023-02743-8 PMC10858824

[36] Y. Liu , L. Shi , W. Qiu , Y. Shi , Mol. Cell. Toxicol. 2022, 18, 509. 35035494 10.1007/s13273-021-00219-5 PMC8744019

[37] T. M. T. Truong , S. H. Seo , S. Chung , I. Kang , J. Nutr. Biochem. 2023, 112, 109204. 36400112 10.1016/j.jnutbio.2022.109204

[38] N. Wittmann , A.‐K. Behrendt , N. Mishra , L. Bossaller , A. Meyer‐Bahlburg , Cells 2021, 10, 2880. 34831104 10.3390/cells10112880 PMC8616555

[39] E. H. Lee , J. H. Shin , S. S. Kim , S. R. Seo , Cells 2021, 10, 2327.34571975

[40] M. Kinra , A. Joseph , M. Nampoothiri , D. Arora , J. Mudgal , Eur. J. Pharm. Sci. 2021, 157, 105637. 33171231 10.1016/j.ejps.2020.105637

[41] X. Wang , Z.‐F. Zhang , G.‐H. Zheng , A.‐M. Wang , C.‐H. Sun , S.‐P. Qin , J. Zhuang , J. Lu , D.‐F. Ma , Y.‐L. Zheng , Molecules 2017, 22, 1315.28786950

[42] M. D. Molla , Y. Akalu , Z. Geto , B. Dagnew , B. Ayelign , T. Shibabaw , J. Inflamm. Res. 2020, 13, 749. 33116753 10.2147/JIR.S277457 PMC7585796

[43] Q. Shan , Y. Zheng , J. Lu , Z. Zhang , D. Wu , S. Fan , B. Hu , X. Cai , H. Cai , P. Liu , F. Liu , Food Chem. Toxicol. 2014, 69, 339. 24795233 10.1016/j.fct.2014.04.033

[44] S. Tedesco , F. de Majo , J. Kim , A. Trenti , L. Trevisi , G. P. Fadini , C. Bolego , P. W. Zandstra , A. Cignarella , L. Vitiello , Front. Pharmacol. 2018, 9, 71. 29520230 10.3389/fphar.2018.00071 PMC5826964

[45] C. R. Nascimento , N. A. Rodrigues Fernandes , L. A. Gonzalez Maldonado , C. Rossa Junior , Biochem. Biophys. Rep. 2022, 32, 101383. 36420419 10.1016/j.bbrep.2022.101383 PMC9677084

[46] D. Mueller , K. Jung , M. Winter , D. Rogoll , R. Melcher , E. Richling , Food Chem. 2017, 231, 275. 28450007 10.1016/j.foodchem.2017.03.130

