# Supplementary material for: Acylated Anthocyanins From Black Carrots and Their Related Phenolic Acids Diminish Priming and Activation of the NLRP3 Inflammasome in THP‐1 Monocytes
Source: Mol Nutr Food Res. 2024 Oct 19;68(22):2400356. doi: 10.1002/mnfr.202400356 (PMC11605781; doi:10.1002/mnfr.202400356)
Supplement: Supplementary file 1 — Supporting Information [file MNFR-68-2400356-s001.pdf]

Supporting Information

Acylated anthocyanins from black carrots and their related phenolic acids diminish priming and activation of the NLRP3 inflammasome in THP-1 monocytes

Inken Behrendt[1*], Katharina Becker[2], Christof Björn Steingass[2], Ralf Schweiggert[2], Gabriela Michel[3,4], Elvira Friedrich[1], Daniela Grote[1], Zoe Martin[1], Hanna Pauline Dötzer[1], Mathias Fasshauer[1], Martin Speckmann[3,4], and Sabine Kuntz[1]

[1]Institute of Nutritional Science, Department of Nutritional Science, Justus-Liebig-University Giessen, Giessen, Germany

[2]Chair of Analysis and Technology of Plant-based Foods – Focus on Beverages, Department of Beverage Research, Geisenheim University, Geisenheim, Germany

[3]Institute for Clinical Immunology, Transfusion Medicine and Hemostaseology, Department of Medicine, Justus-Liebig-University Giessen, Giessen, Germany

[4]Flow Cytometry Core Facility, Department of Medicine, Justus-Liebig-University Giessen, Giessen Germany

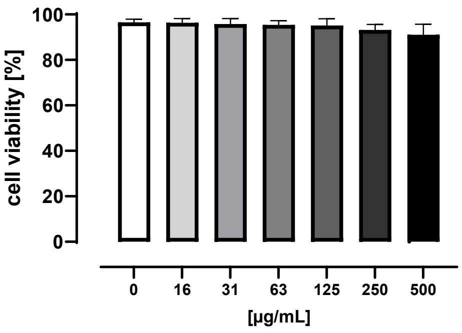

A

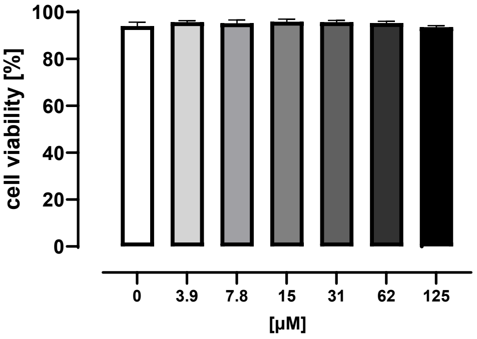

B

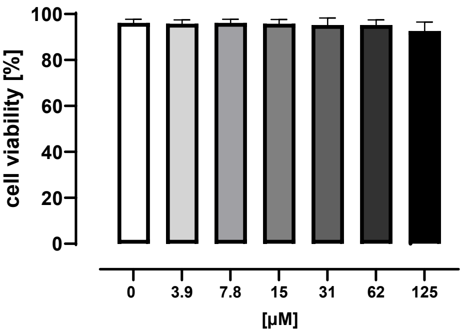

C

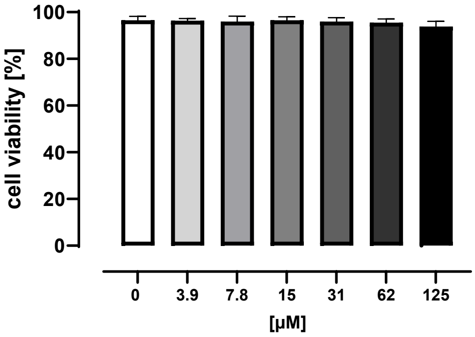

D

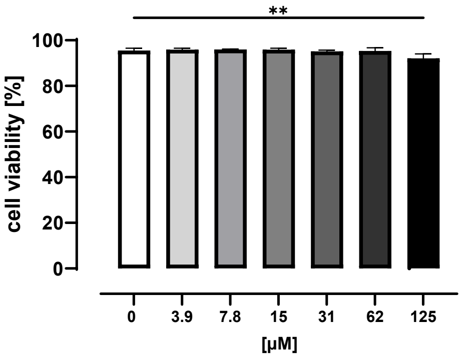

E

Figure S1. Influence of a black carrot extract (BCE) with high amounts of acylated anthocyanins and their related phenolic acids on cell viability of THP-1 monocytes. THP-1 monocytes were incubated with the indicated concentrations of (**A**) the BCE, (**B**) cyanidin-3-glucoside, (**C**) ferulic acid, (**D**) *p*-coumaric acid, or (**E**) sinapinic acid for 24 h before cell viability was assessed as percentage of viable cells by flow cytometry. Significant differences to untreated control cells were determined by one-way analysis of variance (ANOVA) with Dunnett's multiple comparisons test (**$p < 0.01$).

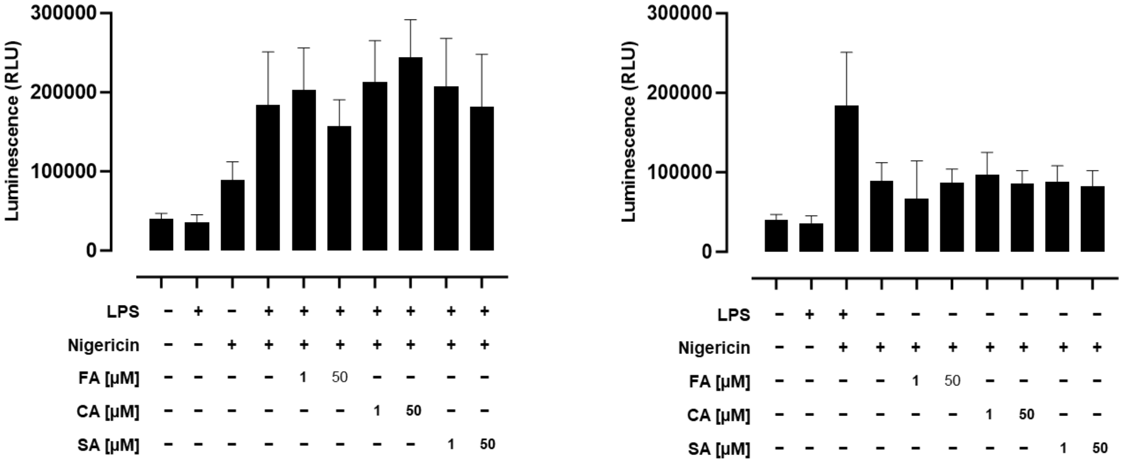

(**A**) (**B**)

Figure S2. Influence of phenolic acids on caspase-1-activity in THP-1 monocytes. THP-1 monocytes were preincubated with the indicated concentrations of the test compounds before the NLRP3 inflammasome was activated. Caspase-1 activity was measured by using a bioluminescent assay and luminescence was measured as relative light unit (RLU). Significant differences to (**A**) LPS stimulated and nigericin-activated cells or (**B**) only nigericin-activated cells were calculated using one-way ANOVA with Dunnett´s multiple comparisons test.

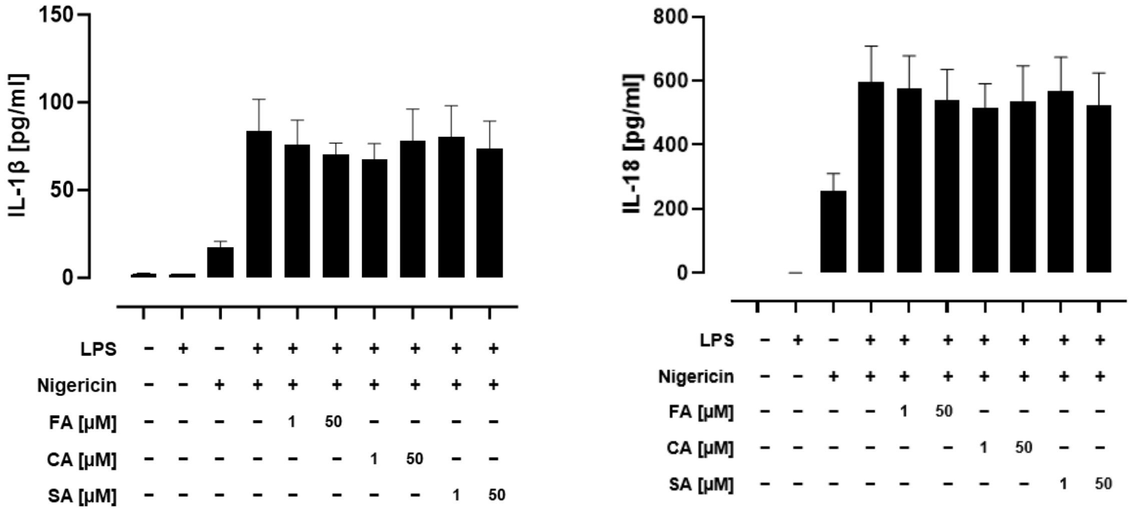

(**A**) (**B**)

Figure S3. Influence of phenolic acids on proinflammatory cytokine release in THP-1 monocytes. THP-1 monocytes were preincubated with the indicated concentrations of the treatment solutions before the NLRP3 inflammasome was activated. Release of (**A**) IL-1β and (**B**) IL-18 into the cell culture supernatant was measured by ELISA. Significant differences to LPS stimulated and nigericin-activated cells were calculated using one-way ANOVA with Dunnett´s multiple comparisons test.
